# Exploring Predictors of Disaster Resilience Among Older Adults: Evidence from a Nationally Representative Survey

**DOI:** 10.1093/geroni/igaf122.094

**Published:** 2025-12-31

**Authors:** Gabriella Meltzer, Kathleen Lynch, Alexis Merdjanoff

**Affiliations:** American University, United States; New York University, New York, New York, United States; New York University, New York, New York, United States

## Abstract

Older adults are the fastest-growing age group in the U.S. and desire to age at home. However, many reside in places increasingly exposed to climate change-fueled disasters. The Aging in Risky Environmental Areas (AREA) Study is a nationally representative survey of community-dwelling adults ages 50+ queried in November 2022 on aging, disaster exposure, and climate change. We conducted bivariate analyses and multivariate linear regression to examine the association between disaster exposure in the past 3 years and scores on the Disaster Adaptation and Resilience Scale (DARS), adjusting for sociodemographics and healthy aging indicators. Among 1,504 participants, 40.3% were exposed to ≥ 2 climate-related disasters. Total disaster exposure (p = 0.002), any indirect exposure (p < 0.001), and any direct exposure (p = 0.005) were all negatively associated with total disaster resilience. Total disaster exposure was also negatively associated with DARS subscales of social resources (p = 0.003), distress regulation (p < 0.001), and optimism (p = 0.006). In the final model, disaster exposure was not associated with resilience. Several healthy aging variables were associated, including place attachment (b = 3.28, p < 0.001); time spent on social and leisure activities (b = 1.23, p < 0.001); loneliness (b = -0.47, p < 0.001); cognitive impairment (b = -8.28, p < 0.001); depression (b = -5.47, p = 0.002); and psychological distress (b = -0.88, p < 0.001). Results show that while disaster exposure is associated with worse resilience, healthy aging can supersede this while facing more frequent and intense climate-related disasters.

